# Was it a HIIT? A process evaluation of a school-based high-intensity interval training intervention

**DOI:** 10.1186/s12966-024-01599-2

**Published:** 2024-04-29

**Authors:** Stephanie L. Duncombe, Alan R. Barker, Lisa Price, Jacqueline L. Walker, Jodie L. Koep, James Woodforde, Michalis Stylianou

**Affiliations:** 1 School of Human Movement and Nutrition Sciences, The University of Queensland, Saint Lucia, QLD 4072 Australia; 2 Children’s Health and Exercise Research Centre, Public Health and Sports Sciences, University of Exeter Medical School, Faculty of Health and Life Sciences, University of Exeter, Exeter, UK

**Keywords:** Child, Implementation, Physical education, Fidelity, Enjoyment

## Abstract

**Background:**

Despite a growing body of research investigating high-intensity interval training (HIIT) in schools, there are limited process evaluations investigating their implementation. This is concerning because process evaluations are important for appropriately interpreting outcome findings and augmenting intervention design. This manuscript presents a process evaluation of *Making a HIIT*, a school-based HIIT intervention.

**Methods:**

The *Making a HIIT* intervention spanned 8 weeks and was completed at three schools in Greater Brisbane, Australia. Ten classes (intervention group) completed 10-min teacher-led HIIT workouts at the beginning of health and physical education (HPE) lessons, and five classes (control group) continued with regular HPE lessons. The mixed methods evaluation was guided by the Framework for Effective Implementation by Durlak and DuPre.

**Results:**

*Program reach*: Ten schools were contacted to successfully recruit three schools, from which 79% of eligible students (*n* = 308, $\overline{{\text{x}} }$ age: 13.0 ± 0.6 years, 148 girls) provided consent. *Dosage*: The average number of HIIT workouts provided was 10 ± 3 and the average number attended by students was 6 ± 2. *Fidelity*: During HIIT workouts, the percentage of time students spent at ≥ 80% of maximum heart rate (HR_max_) was 55% (interquartile range (IQR): 29%—76%). *Monitoring of the control group*: During lessons, the intervention and control groups spent 32% (IQR: 12%—54%) and 28% (IQR: 13%—46%) of their HPE lesson at ≥ 80% of HR_max_, respectively. *Responsiveness*: On average, students rated their enjoyment of HIIT workouts as 3.3 ± 1.1 (neutral) on a 5-point scale. *Quality*: Teachers found the HIIT workouts simple to implement but provided insights into the time implications of integrating them into their lessons; elements that helped facilitate their implementation; and their use within the classroom. *Differentiation*: *Making a HIIT* involved students and teachers in the co-design of HIIT workouts. *Adaption*: Workouts were modified due to location and weather, the complexity of exercises, and time constraints.

**Conclusion:**

The comprehensive evaluation of *Making a HIIT* provides important insights into the implementation of school-based HIIT, including encouragings findings for student enjoyment and fidelity and recommendations for improving dosage that should be considered when developing future interventions.

**Trial Registration:**

ACTRN, ACTRN12622000534785, Registered 5 April 2022 – Retrospectively registered.

**Supplementary Information:**

The online version contains supplementary material available at 10.1186/s12966-024-01599-2.

## Background

Schools are uniquely positioned to support physical activity interventions due to their ability to reach a large proportion of children and adolescents, their existing infrastructure, and staff who can be trained to help implement such interventions [1, 2]. However, schools also present notable challenges, including time constraints and curriculum requirements, which need to be considered when planning interventions [1, 3]. School-based research commands the literature on adolescent physical activity promotion efforts, although contemporary reviews indicate school-based interventions have had minimal success at increasing physical activity levels [2, 4] and some success at improving cardiorespiratory fitness and cognition, with considerable heterogeneity within relevant findings [5–7].

A major influencing factor for the success of school-based physical activity interventions is implementation [2], defined as the process of integrating an intervention within a particular setting [8]. Implementation can be monitored using process evaluations [9], which provide insights into why and how an intervention succeeds (or fails) to accomplish its intended outcomes [10]. A highly used evaluation framework within physical activity literature is Durlak and DuPre’s Framework for Effective Implementation, which includes eight dimensions derived from a comprehensive and rigorous review of over 500 health promotion interventions for children and adolescents [11, 12]. A systematic review applying this framework to school-based physical activity interventions noted that school and teacher level implementation was most often assessed solely on dose received by students [9]. Consequently, the authors called for comprehensive evaluations that include other factors crucial for successful implementation, such as intervention quality and fidelity [9]. Real-world implementation of school-based physical activity interventions has thus far been limited, with available results indicating poorer effectiveness of these studies compared to efficacy trials [3]. Comprehensive process evaluations can help understand discrepancies between efficacy and effectiveness trials, contributing to the enhancement of implementation, adoption, and sustainability of school-based physical activity interventions [3].

High intensity interval training (HIIT) is gaining popularity as an intervention approach in school-based physical activity research. This can be attributed to its similarity to children’s intermittent patterns of physical activity and research associating higher intensity activities with lower cardiometabolic risk [13, 14]. Literature on school-based HIIT interventions has demonstrated positive outcomes for cardiorespiratory fitness, body composition, and blood biomarkers [15–17]. However, systematic reviews have noted high levels of heterogeneity in the assessed outcomes and a paucity of information related to process outcomes [15, 18]. A recent paper on scaling HIIT programs for adolescents highlighted that designing these interventions based on implementation frameworks is critical for their success and called for research investigating their feasibility and acceptability [19]. Two school-based HIIT interventions have previously completed process evaluations, noting that overall, the interventions were well-received [20, 21]. However, the authors noted challenges related to students achieving high-intensity thresholds [20] and recommended including a wider range of exercise activities in future programs [21].

The *Making a HIIT* study is a real-world school-based HIIT intervention adapted for implementation at three separate schools using HIIT workouts co-designed at each school [22]. Teachers led the intervention within Year 7 and 8 Health and Physical Education (HPE) lessons and adapted the intervention to fit the school and class context [22]. The aim of this paper was to comprehensively evaluate the implementation of the *Making a HIIT* study using the Framework for Effective Implementation [11].

## Methods

The *Making a HIIT* study has previously been described in detail in a protocol paper (Trial Registration: ACTRN12622000534785) [22]. A brief overview of the study is provided below.

### Recruitment

Schools in Greater Brisbane, Australia were invited to participate in *Making a HIIT* through purposeful sampling, with the aim of recruiting three schools. Years 7 and 8 (students aged 12 – 14 years) were identified as preferred years for the study as they included relevant HPE curriculum content descriptions, including designing fitness plans and modifying systems to increase enjoyment and success [23]. Participating classes were decided collaboratively by the head of HPE department, individual teachers, and researchers. Students in participating classes were excluded if they had any medical condition or injury that prevented them from participating in HIIT or if they were unable to complete the study measures. The first school recruited served as a pilot school and only half the number of classes were recruited in this school.

The study was approved by The University of Queensland’s human research ethics committee (Project: 2020/HE002444) and education organisations as necessary. Informed consent was collected from school gatekeepers (principals) and teachers. Informed assent and consent were obtained from students and their parents / guardians, respectively.

### Intervention

The *Making a HIIT* study consisted of two phases [22]. In phase one, students co-designed HIIT workouts with their class, teacher, and a researcher as part of their HPE curriculum through an iterative process. The process and the results of this phase have previously been published [24]. In phase two, which employed a quasi-experimental design, co-designed HIIT workouts were used in an intervention. Recruited classes were assigned to one of three groups: 1) co-design group, who co-designed the HIIT workouts and subsequently used them in HPE for a term; 2) HIIT only group, who used the HIIT workouts in HPE for a term but were not involved in the co-design process; and 3) control group, who continued with normal HPE lessons. The intervention spanned 8-weeks of the 10-week term. During the 8-weeks, classes in Group 1 and Group 2 completed a teacher-led 10-min HIIT workout at the start of their HPE lessons before continuing their lesson as normal. The workouts contained a variety of aerobic and resistance exercises and had themes focused on specific sports, locations, muscle activation, or working with friends (see Additional file 1) [24]. To lead the workouts, teachers were provided with a laminated booklet that included the workouts for each week. Prior to the intervention, a researcher met with the teachers to explain and demonstrate the workouts, clarify certain exercises, and answer any questions they had. Schools one and three completed HIIT workouts in both theory and practical lessons, while school two only used HIIT workouts in practical lessons. Theory lessons were completed in a classroom setting, while practical lessons were completed on courts, in halls, or on fields. Sessions completed in the classroom were completed using the standard classroom layout with no movement of chairs or tables. Students were encouraged to provide maximal effort during the ‘work’ periods of HIIT workouts by both the teacher and researcher. Students in Group 3 continued with regular HPE lessons. A researcher was present during the practical lessons for all three groups to administer heart rate (HR) monitors but was not involved in leading the HIIT workouts.

### Theoretical basis

*Making a HIIT* was guided by two theories: the theory of expanded, extended, and enhanced opportunities and self-determination theory [25–27]. The theory of expanded, extended, and enhanced opportunities suggests that more opportunities (expanded), more time for the opportunities (extended), and higher quality opportunities (enhanced) for physical activity will result in higher activity levels [25]. *Making a HIIT* aimed to enhance HPE lessons by introducing curriculum content that targeted high-intensity physical activity and implementing co-design to enhance student input and engagement. Further, it included components to support students’ three basic psychological needs (autonomy, competence, and relatedness) described within self-determination theory, such as the co-design process, exercise modifications, and partner and group workouts.

### Measures collected

To evaluate the implementation of *Making a HIIT*, we collected data aligned with the eight dimensions from the Framework for Effective Implementation [11] as described below.

*Program Reach.* Information was recorded about the number of: 1) schools contacted until three (one co-educational school, one all-boys school, one all-girls school) were successfully recruited; 2) students who received information about the study; and 3) students who consented to participate.

*Dosage.* The researcher and participating teachers recorded information about the number of sessions delivered and number of sessions attended by students.

*Fidelity.* HR data were collected using a HR monitor (Polar H10, Polar Electro, Finland) and Polar GoFit software (https://polargofit.com/) during the HIIT workouts in practical lessons for Groups 1 and 2. Extracted data included students’ average and peak HRs, and time spent in various deciles of maximum HR (HR_max_) (e.g., 50–59%, 60–69%, 70–79%, 80–89%, and 90%—100%). Students’ HR_max_ had previously been determined during a 20 m shuttle run test at baseline or where students did not complete the shuttle run, an age predicted maximum was used. A detailed description of the methods used to quantify HR for *Making a HIIT* is published elsewhere and includes further quantification methods (e.g., the number of students obtaining thresholds and consideration of various thresholds) [28]. For this paper, we report students’ average HR for the HIIT workout (including bouts of work and rest) and the average time that students spent above 80% of their HR_max_. Additionally, students completed the Omnibus Scale of Perceived Exertion immediately after each HIIT workout (ranging from 0 [not tired at all] to 10 [very, very tired]) in both practical and theory lessons to provide a sessional rating of perceived exertion (RPE) [29]. In our study, the correlation coefficient between HR and sessional RPE in practical lessons was 0.39 [28].

*Monitoring of the control group.* For Groups 1 and 2, HR data during the remainder of the HPE lesson was collected using the same methods detailed above in *fidelity*. For Group 3, HR data were recorded for the entire HPE lesson. The researcher recorded the theme of each class’s HPE unit for the term, and the activities completed during the practical lessons in a field note diary.

*Quality of implementation.* A semi-structured interview with each teacher was conducted at the end of the intervention to discuss implementation (see Additional file 2 for question guide used). Interviews were led by the researcher involved in the intervention in conjunction with another researcher. Comprehensive notes were taken during all interviews. Additionally, in school three, interviews were audio recorded and subsequently transcribed. Interviews in the other two schools were not audio recorded due to different school board research requirements.

*Quality of workouts.* The HR and RPE data described above in the *fidelity* section were used to assess the quality of the workouts. The intensity elicited during the HIIT workouts used in practical lessons was examined using both HR and RPE data, and the intensity for the HIIT workouts used during theory lessons (classroom-based) was examined using RPE data.

*Responsiveness.* To assess enjoyment, students responded to “I enjoyed participating in today’s HIIT session” on a 5-point Likert scale ranging from strongly disagree (1) to strongly agree (5) immediately after each workout. A similar question has been used in previous school-based HIIT work to understand student satisfaction [30]. To assess positive and negative affect during HIIT, students in schools two and three completed two scales [31] after completing the first and last HIIT workouts. The positive affect scale included 5 items (proud; satisfied; happy; excited; and relaxed), and the negative affect scale included 4 items (unhappy; nervous; guilty; and angry). Students were asked to rate each item on a 5-point Likert scale ranging from not at all (1) to extremely (5), using the prompt “During this HIIT workout, I felt…”. These scales have previously been used to assess positive and negative affect toward sport in children aged 8 – 13 years with reliability coefficients of 0.75 and 0.78, respectively [31]. Finally, responsiveness was also informed by the semi-structured interviews described above in the *quality of implementation* section.

*Differentiation.* No data were collected during the intervention for the differentiation dimension. The uniqueness of the study was determined during the design of *Making a HIIT* [22] based on a systematic review and meta-analysis on school-based HIIT conducted by the authors [15].

*Adaption.* The researcher kept a field note diary documenting any modifications made to the HIIT workouts by teachers or to the intended HIIT workout schedule, accompanied by information about the reasons for the modifications. This was also informed by the semi-structured interviews with teachers.

### Data analysis

Quantitative data were collected for *program reach*, *dosage, fidelity, quality, responsiveness,* and *monitoring of the control group*. Data were analysed using R (Version 3.6.2; The R Foundation for Statistical Computing, Vienna, Austria) with alpha set to 0.05. Data were assessed for normality using a Shapiro–Wilk test and descriptive statistics were reported. Data from all three schools were combined for reporting the results unless a more nuanced presentation was warranted.

Qualitative data were collected for *quality*, *responsiveness* and *adaption*. A thematic analysis was conducted using the transcripts and sets of notes collected during interviews [32, 33]. All recorded interviews were transcribed verbatim within three days of completion by the first author to increase familiarity with the data. The interview notes were digitised within a week of completing the interviews. Any personal or identifiable information was deleted prior to analysis. After familiarisation with the data, a deductive approach, which focused on the semantic (explicit) meaning of the text, was used to create codes related to the three relevant dimensions by two authors using NVivo (Version R1) [33]. The first author developed the themes based on the codes and all authors reviewed and refined the themes until consensus was reached.

## Results

Three schools from varied backgrounds (co-educational government school; all-girls non-government school; all-boys non-government school) participated in *Making a HIIT* (Table 1). In total, 8 teachers (75% female) and 10 classes (222 students; 46% female) participated in the intervention. The control group included 3 teachers (33% female) and 5 classes (86 students; 52% female). The findings of this study are summarised in Table 2 and extended below according to the eight dimensions of the Framework for Effective Implementation.

**Table 1:** Participating school and class characteristics

School	School Type	Students	ICSEA percentile^a^	Background Language Other than English^a^	Class	Group	Year Level	Average Age (years)	Number of students (% female)
One	State	Co-educational	41%	42%	A	Co-Design	8	13.3 ± 0.3	25 students (44%)
					B	HIIT Only	8	13.3 ± 0.3	12 students (50%)
					C	Control	8	13.2 ± 0.4	12 students (50%)
Two	Independent	Boys Only	87%	24%	D	Co-Design	7	12.6 ± 0.3	25 students (0%)
					E	Co-Design	7	12.5 ± 0.3	24 students (0%)
					F	HIIT Only	8	13.6 ± 0.4	26 students (0%)
					G	HIIT Only	8	13.7 ± 0.3	24 students (0%)
					H	Control	8	13.5 ± 0.2	13 students (0%)
					I	Control	8	13.5 ± 0.4	22 students (0%)
Three	Catholic Education	Girls Only	65%	5%	J	Co-Design	8	13.3 ± 0.3	23 students (100%)
					K	Co-Design	8	13.4 ± 0.3	25 students (100%)
					L	HIIT Only	7	12.4 ± 0.3	20 students (100%)
					M	HIIT Only	7	12.5 ± 0.3	18 students (100%)
					N	Control	7	12.4 ± 0.5	21 students (100%)
					O	Control	7	12.3 ± 0.4	18 students (100%)

^a^The values presented in the school information columns were acquired from myschool.edu.au and are based on 2021/2022 data.* ICSEA *Index of Community Socio-Educational Advantage, which is generated based on family background data that is highly correlated with student performance

**Table 2:** Summary of Durlak and DuPre’s Framework for Effective Implementation dimensions and their indicators and relevant results within the *Making a HIIT* study

Dimension	Definition	Indicator	Result
Program Reach	The rate of involvement and representativeness of participants	Number of schools contacted to recruit three	10
		Number of consenting students	308 / 388 (79%)
Dosage	The amount of the intervention that was delivered	Number of sessions provided by teachers (Theory and practical lessons combined)	School one: 14 ± 3; School two: 9 ± 2; School three: 8 ± 1
	The amount of the intervention that was received	Number of sessions attended by students (Theory and practical lessons combined)	School one: 12 ± 3; School two: 6 ± 2; School three: 6 ± 2
Fidelity	The extent to which the intervention was completed as intended	Average heart rate	161 ± 16 bpm (79% ± 8% of HR_max_)
		Peak heart rate	188 ± 13 bpm (92% ± 6% of HR_max_)
		Percent of time above 80% of HR_max_	55% (IQR: 29%—76%)
		Percent of students with average heart rate above 80% of HR_max_	51% (IQR: 31%—67%)
		Average RPE	In practical sessions: 6 ± 2 In theory sessions: 4 ± 2
Monitoring of control group	Describing the nature and the amount of high-intensity exercise received by this group	Percent of time above 80% of HR_max_	Intervention group during lessons: 32% (IQR: 12%—54%) Control group during lessons: 28% (IQR: 13%—46%)
		Average heart rate	Intervention group during lessons: 75% ± 8% of HR_max_ Control group during lessons: 73% ± 8% of HR_max_
Quality	How well different intervention components were conducted	Average workout heart rate	In practical sessions: 78% ± 4% of HR_max_
		Average workout RPE	In practical sessions: 6 ± 2 In theory sessions: 4 ± 0.5^a^
		Implementation of workouts	Three themes identified: 1) scheduling and time implications of the workouts; 2) facilitation of the workouts; and 3) use of the HIIT workouts within the classroom
Responsiveness	The degree to which the intervention stimulates the interest or holds the attention of participants	Student enjoyment	3.3 ± 1.1 out of 5 (Neutral rating)
		Student affect	Positive affect: 3.0 (IQR: 2.2 – 3.6) Negative affect: 1.5 (IQR: 1.0 – 2.0)
		Student engagement	Two themes identified: 1) engagement over time and 2) elements affecting engagement
		Teacher intent to continue using HIIT	Two themes identified: 1) how they might implement HIIT in the future and 2) curriculum integration
Differentiation	The extent to which an intervention’s theories and practices can be distinguished from other interventions	Uniqueness of the study	Engagement of end-users Integration of the study within the school curriculum
Adaption	Changes made in the original intervention during implementation	Intervention modifications by teachers	Sessions missed due to schedule changes (assemblies, assessments, holidays)
		Workout modifications by teachers	Changes due to availability and quality of space for workouts Equipment removed due to time required to set up (skipping ropes, soccer balls) Simplification of workouts (group exercises removed due to time required to form teams; short intervals combined)

A summary table of the eight dimensions and their definitions from the framework by Durlak and DuPre [11]. The indicators and results from the *Making a HIIT* study are presented for each dimension*.* Further detail for each outcome is provided in text and figures

Quantitative data were reported as frequencies, mean ± standard deviation, or median (interquartile range) based on normality of the data

*HR* Heart rate, *HR*_*max*_ Maximum heart rate, *IQR* Interquartile range, *RPE* Rating of perceived exertion

^a^Provided to one decimal place to indicate the existence of variation within the data

*Program Reach.* During recruitment*,* seven schools declined to participate, one co-educational school and six all-girls schools. Reasons for not participating included no one to champion the project, reluctance to alter scheduled HPE units, or hesitancy to measure body weight. Overall, 79% of eligible students consented but this differed across schools (65% in school one; 84% in school two; 81% in school three). The percentage of students that provided consent in Group 1 (co-design), Group 2 (HIIT only), and Group 3 (control) were 94%, 78%, and 65%, respectively.

**Fig. 1:**
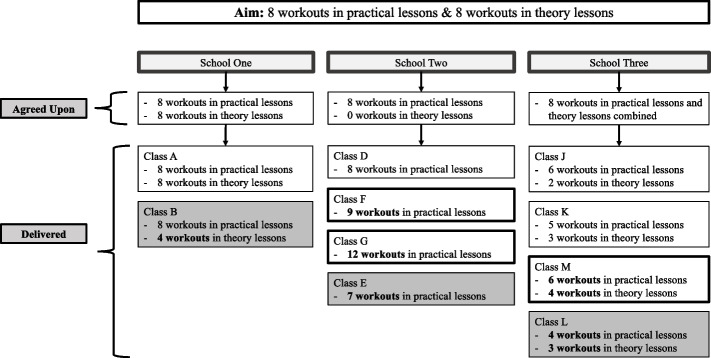
The intended dosage of HIIT for *Making a HIIT*, the dosage of HIIT each school agreed to provide, and the actual dosage of HIIT provided in each class that participated within the study. Within the dosage provided by each class, outlined squares indicate classes where teachers provided a dosage greater than the agreed upon amount and shaded squares indicate classes where teachers provided a dosage less than the agreed upon amount. HIIT = high-intensity interval training

**Fig. 2:**
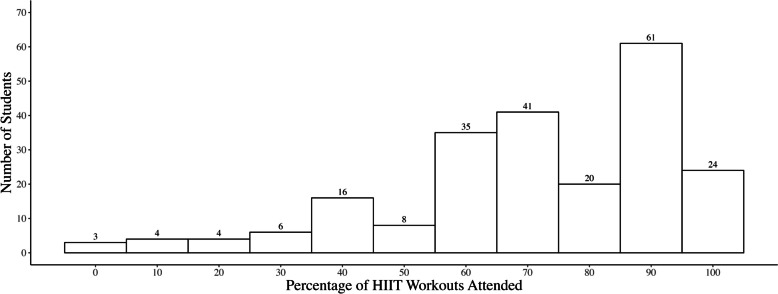
A histogram of the percentage of workouts that each student attended during the *Making a HIIT* intervention. HIIT = high-intensity interval training

*Dosage – HIIT workouts provided.* The agreed upon dosage varied across schools due to curricular demands and perceived ability to perform HIIT within the classroom (Fig. 1). In three of the ten classes, teachers did not provide the agreed upon dosage of HIIT; however, in another three classes the dosage provided was greater than agreed. *Dosage – HIIT workouts attended.* The number of workouts attended by students also varied (Fig. 2). The number of students who attended all the HIIT workouts ranged between 11 and 13% across schools. The percentage of students who attended 80% or more of the workouts provided was 83%, 46%, and 36% in schools one, two, and three, respectively. The main reasons for missing HIIT workouts were: 1) policies that required specific uniforms for participation in HPE; and 2) policies requiring students to finish assignments prior to partaking in practical HPE lessons.

*Fidelity.* The fidelity of the *Making a HIIT* intervention has been previously published in detail [28]. During HIIT workouts in practical lessons, the mean average HR was 79% ± 8% and the mean peak HR was 92% ± 6% of student’s HR_max_. On average across the workouts, 51% of students in a class had an average HR equal to or greater than 80% of their maximum and spent 55% (IQR: 29% – 76%) of time with an average HR equal or above 80% of their HR_max_. The average sessional RPE for the HIIT workouts during practical sessions was 6 ± 2 (Tired), whereas in theory sessions it was 4 ± 2 (Getting more tired).

*Monitoring of the control group.* The HPE units completed by participating classes during the intervention varied between schools and year levels, and were recorded due to the influence that they may have on HR during the lessons. In school one, all three classes of students were completing an “invasion games” unit predominately related to basketball and rugby drills, and small games. In school two, all classes were completing a unit on Australian touch football. In school three, the year seven classes (Group 2 and Group 3) were completing an athletics unit, whilst the year eight classes (Group 1) were completing a unit called “strikes and swings” that included games such as dodgeball, horseshoes, and frisbee golf.

**Fig. 3:**
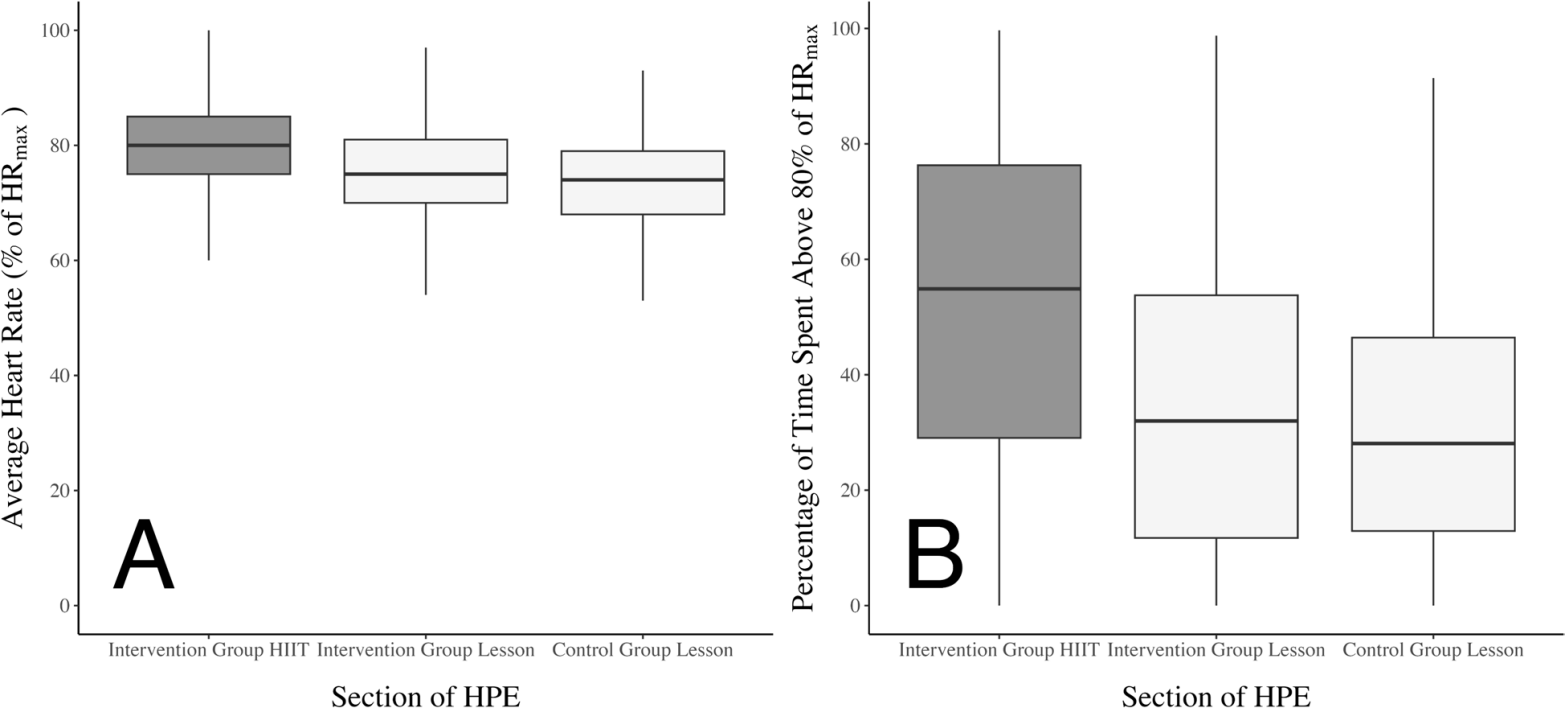
A The average heart rate across all students and sessions during 1) the high-intensity interval training (HIIT) sessions for the HIIT group shown in grey; 2) the HPE lesson for the HIIT group shown in white; and 3) the HPE lesson for the control group shown in white. B The percentage of time students’ heart rate was equal to or greater than to 80% of their maximum heart rate across all students and sessions during 1) the high-intensity interval training (HIIT) sessions for the HIIT group shown in grey; 2) the HPE lesson for the HIIT group shown in white; and 3) the HPE lesson for the control group shown in white. HIIT = high-intensity interval training; HPE = Health and Physical Education

The average HR during lessons for the control group across all three schools was 73% ± 8%, with 28% (IQR: 13% – 46%) of time spent with an average HR equal or above 80% HR_max_. For the intervention group (Groups 1 and 2), the average HR for the remainder of the lesson post HIIT was 75% ± 8%, with 32% (IQR: 12% – 54%) of time spent with an average HR equal or above 80% HR_max_. These data are contrasted to the intervention group HR data during their HIIT workout in Fig. 3. Overall, the intervention group spent 9 min (IQR: 5 – 15 min) with a HR equal or above 80% HR_max_ compared to 6 min (IQR: 3 – 9 min) in the control group during practical lessons. The length of lessons in school one, two, and three were 70 min, 50 min, and 60 min, respectively. The number of minutes with a HR equal or above 80% HR_max_ for the intervention group were 12, 9, and 8 in schools one, two, and three, respectively, compared to 8, 6, and 4 min in the control groups at the three schools.

*Quality of implementation.* Three themes were identified through the thematic analysis for the quality of implementation: 1) the scheduling and time implications of integrating the HIIT workouts into lessons; 2) elements that helped facilitate the implementation of the HIIT workouts; and 3) the use of HIIT during theory lessons within the classroom. Four of eight teachers noted that the workouts took longer than 10 min and occasionally cut into the time scheduled for their planned unit of work due to the additional time required for using the HR monitors, the transition between the activities, or students needing a water break post the workout. However, the teachers found the workouts easy to lead as most of the included exercises were straightforward. They found the laminated booklet of workouts simple to follow and noted that the lack of equipment required made the workouts easier to lead. All the teachers stated that they opted to lead the workouts instead of allowing students to lead the workouts due to time constraints and issues related to limited student confidence, maturity, and level of responsibility. Four teachers noted that the time keeping was challenging with the varying interval lengths and that they struggled to focus on students’ technique and motivation at the same time. The implementation of HIIT within the classroom received mixed feedback from teachers. Teachers in school one perceived HIIT workouts to be a good brain break and beneficial for students, enabling them to settle down. However, teachers in school three struggled with the classroom workouts and identified issues around limited space, calming the students down after the workouts, and lack of suitable uniforms. Specifically discussing uniforms, one teacher highlighted,

“Their uniforms don't really allow for much movement and stretch, and then you're like sweaty in your day uniform, which you have to be in all day. So, knowing girls and everything else, I don't think it's ideal. But it's nice to have that option.”

*Quality of workouts.* In practical lessons, 6 different workouts were completed at school one, 10 at school two, and 8 at school three. On average, these HIIT workouts elicited an average HR of 78% ± 4% and RPE of 6 ± 2. During theory lessons at schools one and three, 9 different workouts were used. Of the 9, 4 were modified versions of practical lesson workouts and 5 were workouts only used in theory class. The average RPE for these workouts was 4 ± 0.5.

**Fig. 4:**
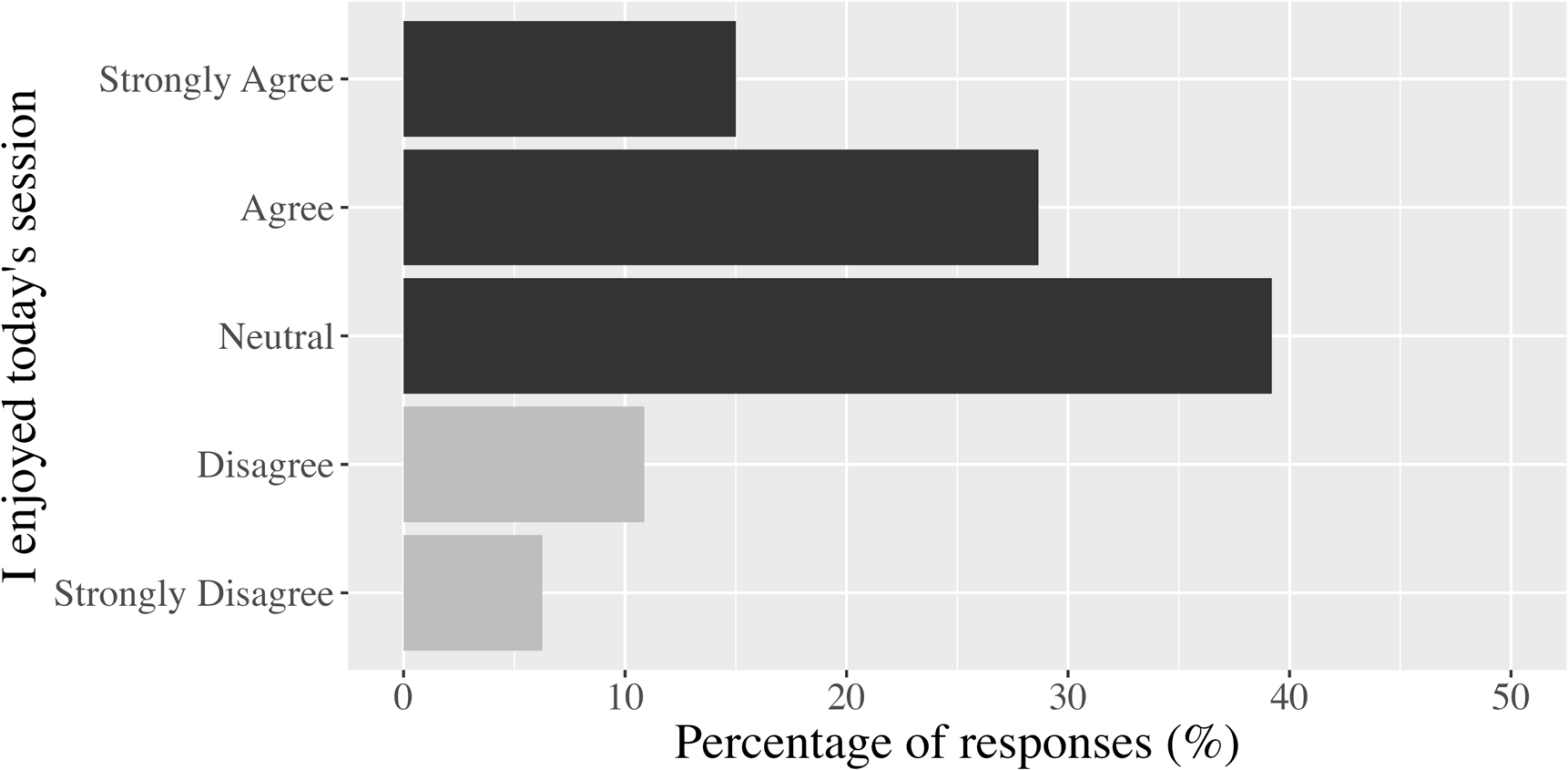
The percentage of responses for each answer to the statement “I enjoyed today’s session…” across all students and high-intensity interval training sessions

**Fig. 5:**
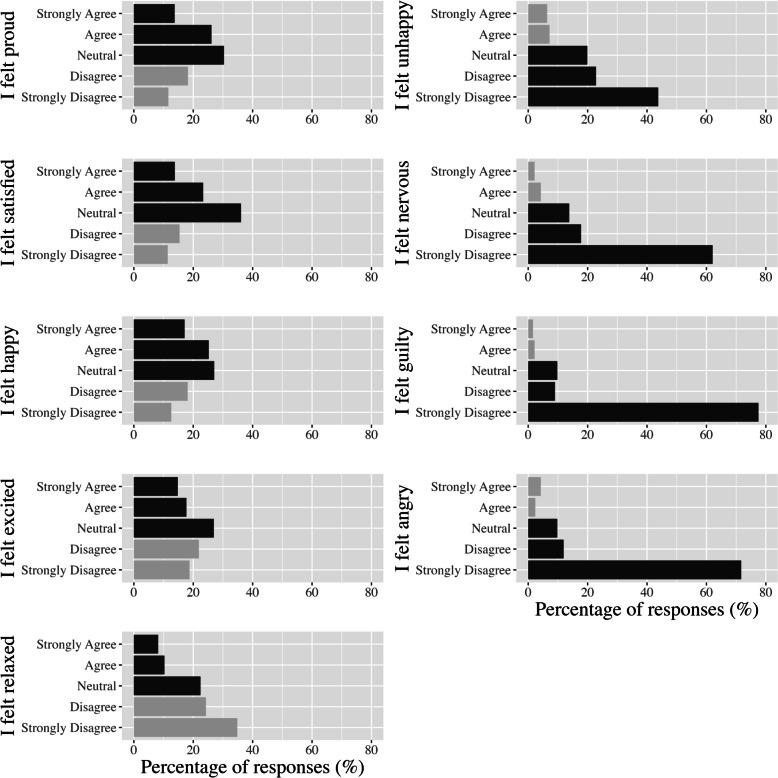
The percentage of responses for each answer across all students and high-intensity interval training sessions for each positive and negative affect prompt

*Responsiveness.* The average rating for session enjoyment for all students and sessions was 3.3 ± 1.1, with 43% of forms indicating “agree” or “strongly agree” (4 or 5) and 17% of forms stating “disagree” or “strongly disagree” (1 or 2) in response to the item “I enjoyed participating in today’s HIIT session” (Fig. 4). Mean enjoyment for HIIT workouts was the same in practical and theory lessons (3.3 ± 1.0). Of the 218 students in the intervention group, 49 (22%) reported an average enjoyment of 4 or 5, while 14 (6%) reported an average enjoyment of 2 or 1. The median (interquartile range) scores for the items included in the positive and negative affect scales were 3.0 (2.2 – 3.6) and 1.5 (1.0 – 2.0), respectively, out of 5. The distribution for each of the 9 items in the affect scales is presented in Fig. 5. Cronbach’s alpha coefficients for the positive and negative scales in *Making a HIIT* were 0.85 and 0.71, respectively.

Two themes relating to responsiveness were identified from the thematic analysis: 1) student engagement; and 2) teacher intent to continue using HIIT. Student engagement was discussed by all eight teachers and included two sub-themes: 1) engagement over time; and 2) elements affecting engagement. Four teachers perceived that student engagement decreased over the intervention as HIIT became less novel and students became more focused on their friends and less on the workout. However, three teachers felt the engagement stayed similar over the eight weeks, noting that students accepted HIIT as part of their routine and had their friends to continue to motivate them. As one of these teachers discussed,

“I think it was almost the same as when they started. I don't think they went negative. I think they stayed the same and, you know, you heard laughter, and they were doing stuff in groups and trying to motivate each other. So, you would say stay the same or even just slightly better.”

Overall, teachers identified various elements that affected student engagement. Three of the five teachers involved in the co-design process noted that it had an impact on engagement and that students tended to be biased towards their own workouts or even workouts made by their classmates. As indicated by one of these teachers stating, “*I think they were more encouraged when it was their groups… that excitement that ‘hey, I designed this program’, but also, ‘that group in my class designed this one.’*”. Certain exercises within the HIIT workouts were perceived to increase engagement, such as exercises that enabled working with friends or that involved competition, while exercises such as burpees, push-ups or exercises requiring use of the floor were noted to decrease engagement. Teachers perceived that the HR monitors, and the presence of the researcher had positive influences on engagement. Finally, teachers noted that students’ general motivation levels within HPE influenced how engaged they appeared in the HIIT workouts, with certain students lacking engagement in both HIIT and HPE.

Two sub-themes were identified within teachers’ intent to continue using the HIIT workouts: 1) curriculum integration; and 2) how they might implement HIIT in the future. All eight teachers noted either a year level or unit that HIIT could fit in in the future. The units they recommended tended to focus on general fitness, while the year level they recommended for the intervention ranged from 5 to 12, with teachers commenting on ties between the curriculum for both general obligatory HPE and senior elective subjects, such as Sport and Recreation. Six teachers discussed that they would continue to use HIIT in their future lessons. Teachers discussed using HIIT as a dynamic warmup in HPE lessons or a classroom break in other subjects. They also indicated that there were aspects within the HIIT workouts that they would continue to use, such as the game-based or fitness-focused exercises, depending on the HPE unit they were completing. For example, one teacher stated,

“I would keep using the partnered activities. Is it possible to pick and choose, I love the workouts. The ones that had mainly partnered activities I would definitely use again. And if it was a time where we had more of a fitness focus, I would lean into more of those that are independent, because fitness is personal.”

*Differentiation.* A point of differentiation for *Making a HIIT* is that it contained involvement from end-users. Teachers were involved in the decision making for the frequency and timing of the HIIT workouts during the intervention. Further, both the teachers and students were involved in co-designing the HIIT workouts used throughout the intervention, which is novel in school-based HIIT research [15]. Lastly, educative outcomes were considered in the design of the intervention and achieved by the students involved in the co-design process [24].

*Adaption.* During the *Making a HIIT* intervention, modifications were occasionally made to workouts. At school one, equipment and relays were removed due to time constraints within the HPE lessons. Consequently, based on this feedback, the co-designed workouts at schools two and three were developed without the use of equipment. To simplify the workouts, teachers sometimes combined two shorter intervals (e.g., 10 s) into one longer interval so they could focus more on the students rather than timekeeping. Further, some of the movements (e.g., burpees, push-ups, squat jumps) were occasionally simplified so students could understand them and perform them effectively. Teachers also sometimes modified the workout due to space availability. For example, this included confining the students to a small area of the field for running based activities or changing exercises such as when, “*we're down on the oval and there is like wet grass or around the courts, and it's too hard to do an [exercise].*”

Modifications were also made to the workouts completed during theory lessons in the classroom. This was done with the researcher and teachers before the intervention began and the updates were included in the final workout booklets provided to the teachers. For school one, this entailed changing running-based intervals to stationary running. In school three, where the uniform included skirts, certain exercises (e.g., floor-based exercises, such as mountain climbers) were modified to enable participation (e.g., standing exercises, such as high-knee running on the spot). Further, on two occasions at school three, the 10-min workout was shortened to 5-min due to the length of time teachers needed to calm the students down and continue the lesson.

Modifications to the frequency and duration of the intervention were also made. In school one, class B had four theory lessons in the computer lab and did not complete the HIIT workouts during those lessons. In schools two and three, assessment, assemblies, and public holidays led to fewer HPE lessons completed over the term and therefore, decreased the dosage of HIIT workouts delivered.

## Discussion

The process evaluation reported in this paper was guided by Durlak and DuPre’s Framework for Effective Implementation and used both quantitative and qualitative data to comprehensively evaluate the *Making a HIIT* intervention [11]. It offers unique insights into the implementation of HIIT workouts within the school setting, which can be used as a lens for examining outcome variables within *Making a HIIT* and to improve future school-based HIIT interventions.

*Making a HIIT* differs from other school-based HIIT interventions due to the engagement of end-users. It was designed with reference to the Australian HPE curriculum content descriptions and was able to elicit educative outcomes through the co-design process to create the HIIT workouts [24]. However, the required engagement by end-users also influenced programme reach. Seven of the ten schools that were approached declined to participate and the most frequently provided reason was because the HPE department was unsure how to fit the co-designing of HIIT workouts within their pre-existing units or was hesitant to replace a unit with the co-design lessons included in *Making a HIIT*. While systematic reviews on HIIT in schools have called for end-user involvement [15, 17], in practice, the amount of end-user input will need to be adaptable at different schools and within different year levels. Designing future studies to accommodate the varying levels of end-user engagement based on school and teacher needs and preferences will be important to consider for improving programme reach. This could include involving students and teachers at a lower level on the participation continuum where instead of co-designing the parameters and exercises of the HIIT workouts, workout frameworks could be provided and modified as needed by the HPE department and individual teachers.

Within *Making a HIIT*, only 26 students (12%) across all three schools attended all the HIIT workouts delivered. This was lower than expected, considering that the HIIT workouts occurred during obligatory lessons. This is also lower than what has been reported by the two other school-based HIIT interventions with process evaluations, with one study reporting a mean attendance of 77% of total sessions and the other stating that students reported completing all three required workouts per week [20, 21]. The low attendance could be due to the three major barriers that presented themselves during the *Making a HIIT* study: uniform policies, curricular demands, and conducting HIIT within the classroom. In school one, where the highest attendance was noted, the uniform policy was not an issue as students were permitted to wear their HPE uniform (i.e., shorts and t-shirts) on days that included either practical or theory HPE lessons. In schools two and three (the all-boys and all-girls schools, respectively), students were required to wear formal uniforms throughout the day, including during theory-based HPE lessons. Although the exercises in the HIIT workouts were adapted to account for this, the uniforms still limited the ability of HIIT to be completed in the classroom. Further, if students did not bring the appropriate practical HPE uniform, they were unable to participate in those HPE lessons either (and therefore, HIIT). Future studies will need to consider varying uniform policies and adapt interventions to meet the needs of individual schools, and if possible, encourage the use of sports uniforms throughout the school day. Available evidence demonstrates that policies that permit students to wear their HPE uniform throughout the day are associated with a significant reduction in sedentary time and non-significant increases to light activity in 8 – 10 year old students [34]. Further, available findings show that most surveyed Australian parents (78%) support policies that enable students to remain in their sports uniforms [35].

In all three schools, curricular demands and related policies led to lower attendance in the HIIT workouts as students needed to prioritise completing schoolwork and assignments that they had missed in theory HPE lessons over partaking in practical HPE lessons. Enabling the delivery of the HIIT workouts at several points in the school day (e.g., lunch, HPE, other lessons) could facilitate enhanced attendance and account for the workouts missed for abovementioned reasons. Previous studies have used HIIT workouts during lunch, before school, and after school [36–38]. However, these studies included small samples that limit the generalisability of the findings and further, as these are discretionary periods, they can potentially lead to the inclusion of only students who are already motivated to be active.

Including HIIT activity breaks in subjects other than HPE should also be considered in the future. However, in the *Making a HIIT* study, classroom-based HIIT received mixed reviews from teachers, with some noting several barriers to its implementation. In school two, for example, classroom size and uniform policies led to the exclusion of classroom-based HIIT. Classroom management was also identified as an issue in school three, with teachers stating they struggled to calm down the students after the workout. However, teachers in school one stated that they found the workouts to have a calming effect on the students. This discrepancy could be due to different teacher-student dynamics or to differences between the student populations at the two schools. Further, the classroom size in school one was larger than school three, which may have facilitated student participation in HIIT. A previous study has successfully used HIIT workouts within the classroom in primary schools in Canada and noted that they were associated with improved off-task behaviour [39]. A systematic review and meta-analysis on physically active lessons found enhanced educational outcomes; however, 39 of the 42 included studies were completed in primary schools and the authors called for further research into active lessons in high/secondary schools [40]. Primary school classrooms tend to be more open with space for play, which could aid in the completion of classroom-based exercise [41]. Future studies on school-based HIIT will need to consider how and when these interventions occur accounting for classroom size, school uniforms, and curricular demands. Enabling flexibility in the delivery of HIIT to account for the variation and unique characteristics within different schools will be important for increasing the dosage delivered and received. Additionally, considering further measures to understand the implementation of these interventions, such as session observations to inform the quality of delivery and student focus groups to further understand responsiveness, should be considered.

While the dosage of HIIT was lower than intended, students had favourable perceptions of the workouts. Students tended to either rate the workouts as enjoyable or neutral, which is similar to other studies that have investigated enjoyment of school-based HIIT in adolescents aged 13 – 18 years [20, 30]. Additionally, very few students reported feeling negative emotions, such as unhappiness (12% of students) or nervousness (6% of students), compared to feeling positive emotions, such as satisfaction (41% of students) and happiness (42% of students), during HIIT. This corroborates previous findings in adolescents where HIIT did not elicit prominent unpleasant feelings during a laboratory-based study [42] and provides further evidence against previous concerns in the literature that HIIT would evoke feelings of displeasure, which could limit engagement and future adherence [43, 44]. Teachers noted that students particularly enjoyed working with their friends, competing against each other, and quick changing intervals. The insight into student engagement was limited to quantitative data from students and teacher qualitative data and could be enhanced by qualitative data from students in future studies for a more nuanced understanding of how to optimise enjoyment of HIIT. However, even without student qualitative data, the evidence is in favour of HIIT eliciting limited negative responses from students.

Some teachers perceived student engagement decreased over time and that students who were unmotivated in general toward HPE tended to be unmotivated towards HIIT. This indicates that future studies will need to consider how to encourage continued engagement in the workouts and how to motivate students who do not enjoy HPE in general. However, the HR data from *Making a HIIT* indicate that students were for the most part completing high-intensity exercise, which is similar to previous school-based HIIT studies [28, 45]. This finding is promising due to the benefits associated with this type of activity, such as improved cardiorespiratory fitness, body composition, and blood biomarkers [14, 15, 46].

In addition to students, teachers also positively responded to the HIIT intervention. Six of eight teachers stated their intent to continue using the workouts in the future in both HPE lessons and other subjects, which is encouraging for the scalability of HIIT within schools. Although, this must be viewed with the knowledge that the researcher involved with the intervention was conducting the interviews, which could have introduced some bias. Teachers commented that the HIIT workouts were able to be adapted, which was an important aspect due to time constraints, scheduling and location changes, and varying levels of class behaviour. This adds to evidence from two previous studies on school-based HIIT where 80% (*n* = 22) and 100% (*n* = 4) of teachers agreed that they intended to use HIIT in their lessons in the future [20, 30]. However, available evidence showcases that while teachers may intend to continue incorporating HIIT, the amount of HIIT does diminish once interventions finish, which is an important consideration for future scale-up [20].

## Conclusion

The comprehensive mixed methods evaluation of *Making a HIIT* provides important insights into the implementation of HIIT within the school setting. The satisfaction of HIIT expressed by students and teachers as well as the overall fidelity of the HIIT workouts are promising for future use of HIIT in this setting. Future studies will need to consider the various options for delivering the HIIT workouts throughout the school day to maximise the dosage received by students and optimise potential health benefits. It will also be important for future studies to make use of dosage and fidelity data within their per-protocol analysis of outcomes to gain greater insights into the effectiveness of HIIT. Additionally, the potential to augment end-user engagement within schools to increase satisfaction without risking decreased programme reach will need to be further investigated. Consultation with teachers and students will enable future studies to minimise barriers, maximise dosage, and increase the positive perceptions of HIIT within the school setting.

### Supplementary Information

**Additional file 1.** Example high-intensity interval training workouts from Making a HIIT. Four examples of the 10-minute high-intensity interval training workouts used within Making a *HIIT*.

**Additional file 2.** Interview guide for discussing the Making a HIIT implementation with teachers. The interview guide used for the semi-structured interviews completed with teachers involved in the implementation of *Making a HIIT*.

## Data Availability

Data are available from the corresponding author on reasonable request.

## Abbreviations

HIIT: High intensity interval training
HPE: Health and physical education
HR: Heart rate
HR_max_: Maximum heart rate
IQR: Interquartile range
RPE: Rating of perceived exertion
