# Supplementary material for: Was it a HIIT? A process evaluation of a school-based high-intensity interval training intervention
Source: Int J Behav Nutr Phys Act. 2024 Apr 29;21:49. doi: 10.1186/s12966-024-01599-2 (PMC11059682; doi:10.1186/s12966-024-01599-2)
Supplement: Supplementary file 1 — Additional file 1. Example high-intensity interval training workouts from Making a HIIT. Four examples of the 10-minute high-intensity interval training workouts used within Making a HIIT. [file 12966_2024_1599_MOESM1_ESM.pdf]

Additional file 1. Example high-intensity interval training workouts from *Making a HIIT*.

Example 1:

DAAL (Theme: Working with Friends)

Equipment: ball

2 x	20s double relay	10s rest
2 x	20s conjoined sit ups	10s rest
40s	star jump	20s rest
40s	squat throw	20s rest
2 x	20s bum kicks	10s rest

Relay = running from opposite lines and high five partner
Sit up = face partner with feet touching, high five at top
Squat throw = squat and throw ball to partner

Example 2:

Ben 10 Fan Club (Theme: Home Workout)

2 x

30s Russian twists	30s rest
35s high knees	25s rest
25s pushups	35s rest
35s bum kicks	25s rest
40s run on spot	20s rest

Example 3:

Kool Kidz (Theme: 80s Aerobics)

Equipment: skipping ropes

2 x

45s	dance boxing (squat, kick, turn around)	15s rest
45s	skipping	15s rest
45s	plank	15s rest
50s	burpee and sprint to line	10s rest
45s	squat (10x) then hold at bottom for 5s	15s rest

Example 4:

[Insert Team Name Here] (Theme: Football Warm-Up)

30s open/close the gate	10s sprint	20s rest
30s high knees	30s rest	
30s pushups	30s rest	

40s bum kicks	20s rest	
20s sprint	20s rest	20s sprint
