# Supplementary material for: Was it a HIIT? A process evaluation of a school-based high-intensity interval training intervention
Source: Int J Behav Nutr Phys Act. 2024 Apr 29;21:49. doi: 10.1186/s12966-024-01599-2 (PMC11059682; doi:10.1186/s12966-024-01599-2)
Supplement: Supplementary file 2 — Additional file 2. Interview guide for discussing the Making a HIIT implementation with teachers. The interview guide used for the semi-structured interviews completed with teachers involved in the implementation of Making a HIIT. [file 12966_2024_1599_MOESM2_ESM.pdf]

Additional file 2. Interview guide for discussing the *Making a HIIT* implementation with teachers.

1. How did you choose to deliver the HIIT workouts and why?
    a. Teacher / student / combination?

2. How did you feel delivering the HIIT workouts?
    a. What worked well?
    b. What did not work well?

3. What, if anything, could we could have done differently to make you feel better equipped to lead the workouts?

4. What, if anything, did you find was beneficial about HIIT workouts for the HPE class?

5. How did the students react to the HIIT workouts?
    a. What did they enjoy?
    b. What did they not enjoy?
    c. What changes, if any, did you notice over the course of the term with regard to the students' engagement in the HIIT workouts?
    d. What differences, if any, did you notice between the students involved in the co-construction process and the students that were not?

6. Which workouts, if any, seemed to work better in HPE class? Why do you think that is?

7. Are there any HIIT workouts (these specific ones or others) that you will continue to use?
